# The correlation between lipoprotein(a) and major adverse cardiovascular events in patients with acute myocardial infarction combined with heart failure with preserved ejection fraction

**DOI:** 10.3389/fcvm.2025.1515916

**Published:** 2025-06-09

**Authors:** Xiaodong Zhang, Nan Niu, Shengqin Yu, Xinxin Zhang, Yanli Zhang, Xuefu Chen, Wenmiao Zhang, Song Yang, Ning Zhang, Yunlong Xia, Ying Liu

**Affiliations:** ^1^ Department of Cardiology, The First Affiliated Hospital of Dalian Medical University, Dalian, China; ^2^ Department of Cardiology, The Second Affiliated Hospital of Dalian Medical University, Dalian, China

**Keywords:** lipoprotein(a), acute myocardial infarction, heart failure, heart failure with preserved ejection fraction, major adverse cardiovascular events

## Abstract

**Aims:**

This study aimed to confirm the correlation between lipoprotein(a) [Lp(a)] and major adverse cardiovascular events (MACE) in patients with acute myocardial infarction (AMI) combined with heart failure with preserved ejection fraction (HFpEF).

**Methods:**

This retrospective study was conducted at the First Affiliated Hospital of Dalian Medical University and included 399 patients who were diagnosed with AMI combined with HFpEF and who were hospitalised and underwent percutaneous coronary intervention (PCI) treatment between January 1, 2018, and January 1, 2023. Based on Lp(a) levels, patients were divided into three tertiles: T1 (≤356 mg/L), T2 [356 mg/L < Lp(a) ≤ 487 mg/L], and T3 (>487 mg/L). The study employed univariate and multivariate Cox regression analysis, subgroup analysis, and receiver operating characteristic (ROC) curve analysis to evaluate the correlation between Lp(a) and MACE.

**Results:**

Compared to the non-MACE group, the MACE group had higher levels of Lp(a) (*P* < 0.001). Tertile-based analysis of Lp(a) levels showed that as Lp(a) increased, the incidence of MACE, rehospitalization due to worsening HF, non-fatal recurrent MI, and unplanned repeat revascularization all increased significantly (all *P* < 0.05). During an average follow-up period of 30.5 months, multivariate Cox regression analysis confirmed that Lp(a) consistently remained an independent predictor of MACE across unadjusted, partially adjusted, and fully adjusted models (all *P* < 0.05). Further component analysis indicated that Lp(a) was significantly associated with cardiac death, rehospitalization due to worsening HF, and non-fatal recurrent MI, with the highest risk observed in the T3 group. Subgroup analysis further demonstrated that the association between elevated Lp(a) and MACE remained statistically significant across various strata (all *P* < 0.05). ROC curve analysis revealed that the area under the curve (AUC) for Lp(a) in predicting MACE was 0.662 (95% CI: 0.607–0.718), which was higher than that of systolic blood pressure (AUC = 0.560) and fasting plasma glucose (AUC = 0.543), but not significantly different from age (AUC = 0.610, *P* = 0.211).

**Conclusions:**

In patients with AMI combined with HFpEF, elevated Lp(a) levels were significantly associated with an increased risk of MACE, and this association remained consistent across multiple subgroups.

## 1 Introduction

Cardiovascular disease (CVD) remains the leading cause of morbidity and mortality globally, and acute myocardial infarction (AMI) represents one of its most critical clinical manifestations. Despite advancements in reperfusion strategies and pharmacologic therapies, both the incidence and long-term prognosis of AMI continue to pose significant clinical challenges. Heart failure (HF) following MI is recognized as a major contributor to reduced quality of life, rehospitalization, and death among affected individuals (1, 2). A systematic review and meta-analysis published in 2023 reported that more than 3 million individuals are diagnosed with acute ST-segment elevation myocardial infarction (STEMI) annually, with approximately 23.3% subsequently developing HF-related complications within the broader coronary artery disease population (3). Lenselink and colleagues noted that despite timely reperfusion in STEMI, HF occurred in 10.9% of cases, with roughly half of these patients retaining normal or mildly reduced left ventricular ejection fraction (LVEF) (1). These observations underscore the need for deeper understanding of post-AMI HF phenotypes, particularly heart failure with preserved ejection fraction (HFpEF), which is increasingly recognized as a distinct and clinically relevant subtype (4).

Well-established risk factors for AMI include dyslipidemia, hypertension, diabetes mellitus, smoking, and obesity. Inadequate control of these factors may exacerbate ventricular remodeling following AMI, increasing the likelihood of HF development. Patients who develop HF after AMI generally exhibit worse outcomes compared to those with isolated AMI. While numerous studies have addressed strategies for HF prevention and treatment post-AMI, limited attention has been given to differentiating between HF subtypes, particularly HFpEF. Though heart failure with reduced ejection fraction (HFrEF) is more commonly observed post-AMI, there is mounting evidence that a substantial subset of patients develops HFpEF—an entity with unique pathophysiological and therapeutic considerations. Consequently, identifying specific risk factors contributing to adverse cardiovascular events in this subgroup is of growing importance.

Among emerging biomarkers of cardiovascular risk, lipoprotein(a) [Lp(a)] has garnered increasing attention. In addition to low-density lipoprotein cholesterol (LDL-C), Lp(a) has been identified as an independent contributor to atherosclerotic cardiovascular disease. Structurally similar to LDL-C, Lp(a) consists of an apolipoprotein B100 molecule and an apolipoprotein(a) component, which resembles fibrinogen and may contribute to thrombogenicity and inflammation. These properties support Lp(a)'s role as a mediator of both atherosclerosis and vascular dysfunction (5). Several studies have also demonstrated strong associations between elevated Lp(a) concentrations and the risk of coronary artery disease, coronary severity, and major adverse cardiovascular events (MACE) (6–8). Despite recent progress in lipid-lowering therapy—such as statins, fibrates, cholesterol absorption inhibitors, and proprotein convertase subtilisin/kexin type 9 (PCSK9) inhibitors—most of these agents have minimal effect on Lp(a) levels. While compounds like niacin and cholesterol ester transfer protein inhibitors may reduce Lp(a), their clinical efficacy in preventing cardiovascular events remains limited (9–11).

However, the relationship between Lp(a) levels and MACE in patients with AMI complicated by HFpEF remains unclear, and relevant studies are scarce. Given the unique pathophysiological features and growing clinical burden of HFpEF, clarifying whether elevated Lp(a) contributes to poor prognosis in this specific population is of significant importance. Therefore, this study aims to investigate the association between Lp(a) levels and the risk of MACE in patients with AMI and HFpEF undergoing percutaneous coronary intervention (PCI), in order to provide novel insights for risk stratification and potential targets for clinical management.

## 2 Methods

### 2.1 Study population

**Figure 1:**
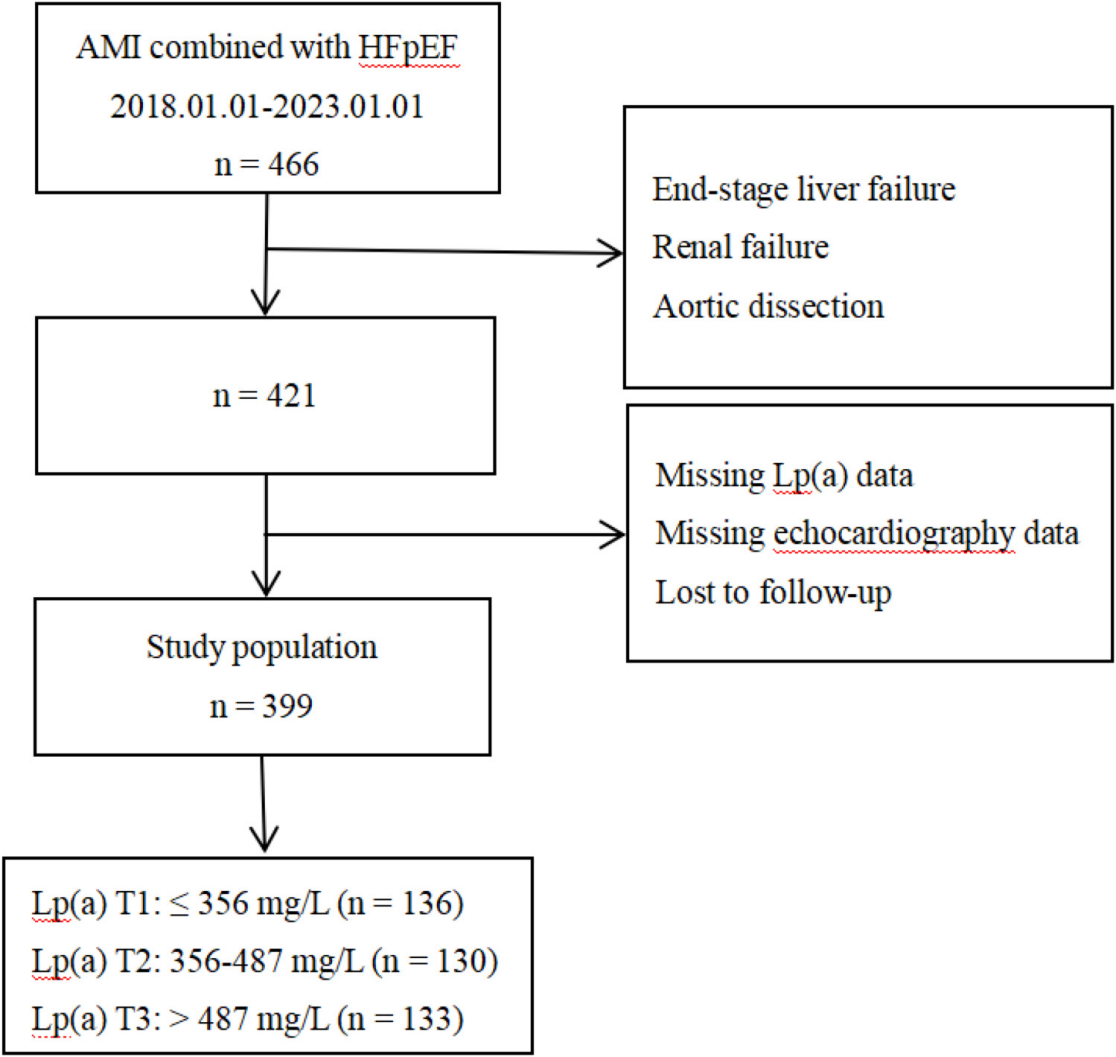
Flowchart of patient selection. Lp(a), lipoprotein(a); AMI, acute myocardial infarction; HFpEF, heart failure with preserved ejection fraction.

This was a single-center retrospective cohort study that included patients who were diagnosed with AMI combined with HFpEF and who were hospitalised and received percutaneous coronary intervention (PCI) at the First Affiliated Hospital of Dalian Medical University between January 1, 2018, and January 1, 2023. After excluding patients with end-stage liver failure, renal failure, aortic dissection, or missing Lp(a) or echocardiography data and those lost to follow-up, a total of 399 patients were included in the final analysis (Figure 1). The study protocol was approved by the Ethics Committee of the First Affiliated Hospital of Dalian Medical University, and all procedures were conducted in accordance with the Declaration of Helsinki and its amendments. Written informed consent was obtained from all participants prior to the collection of clinical data.

### 2.2 Data collection and definitions

All clinical data were collected from the electronic medical records system. The demographic variables included age, sex, smoking and alcohol consumption history, and family history of coronary heart disease (CHD). Smoking was defined as smoking continuously or cumulatively for 6 months or more prior to enrollment. Alcohol consumption was defined as long-term heavy drinking. Data on comorbidities and medications included hypertension, diabetes, stroke, atrial fibrillation (AF), chronic kidney disease (CKD), and the use of antihypertensive, antidiabetic, lipid-lowering, and anticoagulant drugs. Hypertension was diagnosed on the basis of a systolic blood pressure (SBP) ≥140 mmHg and/or a diastolic blood pressure (DBP) ≥90 mmHg in adults (12). The diagnosis of diabetes was based on the presence of symptoms along with a random blood glucose level >11.1 mmol/L, fasting plasma glucose (FPG) ≥7.0 mmol/L, oral glucose tolerance test 2-h glucose >11.1 mmol/L, or hemoglobin Alc (HbA1c) ≥6.5% (13). Stroke was defined as a disorder of cerebral blood circulation caused by either obstruction or rupture of cerebral vessels, resulting in damage to brain tissue function and structure, including ischemic and hemorrhagic stroke (14). AF was defined as a rapid heart rhythm disorder characterised by the loss of atrial electrical activity, leading to rapid and disordered AF waves (15). CKD was defined as chronic structural and functional kidney impairment (history of kidney damage for more than 3 months), including pathological damage with a normal or abnormal estimated glomerular filtration rate (eGFR), abnormalities in blood or urine composition, or unexplained decreased eGFR (<60 ml/min/1.73 m^2^) for more than 3 months, which was considered CKD (16). The anthropometric variables included body mass index (BMI), SBP, DBP, pulse pressure, and heart rate. In this study, the diagnosis of AMI was based on the Fourth Universal Definition of Myocardial Infarction (2018) (17). The diagnosis required fulfillment of the following two key criteria: (1) evidence of myocardial injury, defined as at least one cardiac troponin I or T (cTnI or cTnT) value above the 99th percentile upper reference limit, accompanied by a rising and/or falling pattern; and (2) clinical evidence of myocardial ischemia, demonstrated by at least one of the following: (i) typical ischemic symptoms, such as chest pain, chest tightness, radiating pain, diaphoresis, dyspnea, or nausea; (ii) electrocardiographic (ECG) changes, including new ST-segment elevation or depression, T-wave inversion, new left bundle branch block, or the development of pathological Q waves; (iii) imaging evidence of new loss of viable myocardium or new regional wall motion abnormalities consistent with ischemic etiology; or (iv) identification of an acute coronary artery occlusion, plaque rupture, or thrombus by coronary angiography (17). Furthermore, according to ECG findings, AMI was subclassified into ST-elevation myocardial infarction (STEMI), characterized by persistent ST-segment elevation, and non-ST-elevation myocardial infarction (NSTEMI), which typically presents with ST-segment depression or T-wave changes. The diagnosis of HFpEF was based on the 2021 European Society of Cardiology (ESC) Guidelines for the diagnosis and treatment of HF (18). HFpEF was defined by the presence of the following three criteria: (1) typical signs and/or symptoms of HF, such as dyspnea, fatigue, or reduced exercise tolerance; (2) a LVEF ≥50%; and (3) objective evidence of left ventricular diastolic dysfunction and/or elevated filling pressures. This included structural abnormalities (such as left atrial volume index >34 ml/m^2^, left ventricular mass index ≥95 g/m^2^ in females or ≥115 g/m^2^ in males, or relative wall thickness >0.42), functional abnormalities (such as E/e' ratio >9, tricuspid regurgitation velocity >2.8 m/s, or pulmonary artery systolic pressure >35 mmHg), and elevated natriuretic peptides [N-terminal pro-B-type natriuretic peptide (NT-proBNP) >125 pg/ml or B-type natriuretic peptide (BNP) >35 pg/ml in sinus rhythm; NT-proBNP >365 pg/ml or BNP >105 pg/ml in AF] (18). Patients were diagnosed with HFpEF only if all three criteria were met (18).

Blood biomarkers included kidney function, FPG, HbA1c, lipid profiles, Lp(a), fibrinogen (FIB), D-dimer, high-sensitivity C-reactive protein (Hs-CRP), high-sensitivity-cTnI (Hs-cTnI), and BNP. Kidney function tests included uric acid (UA) and the eGFR, with the eGFR calculated using the Cockcroft-Gault formula (19). Lipid profiles included triglycerides, total cholesterol (TC), high-density lipoprotein cholesterol (HDL-C), LDL-C, apolipoprotein A1 (ApoA1), and apolipoprotein B (ApoB).

The echocardiography data included LVEF, left atrial diameter (LAD), left ventricular end-diastolic diameter (LVDD), interventricular septal thickness (IVSD), and left ventricular posterior wall thickness (LVPW), all measured and recorded by experienced echocardiography physicians using cardiac ultrasound equipment, with LVEF assessed using the biplane Simpson's method.

Procedure-related data included multi-vessel disease, triple-vessel disease, left main (LM), left anterior descending artery (LAD), left circumflex artery (LCX), and right coronary artery (RCA) lesions, as well as stent length and diameter. Multivessel disease was defined as the involvement of two or more coronary arteries with stenosis ≥50%, presenting as diffuse lesions. Three-vessel disease was defined as stenosis ≥50% in all three coronary arteries (RCA, LAD, and LCX), also presenting as diffuse lesions.

### 2.3 Study endpoints and follow-up

This study began follow-up from the patient's first hospitalization day, with follow-up concluding upon patient death or by July 31, 2024. The median follow-up period was 24.6 months. The primary endpoint of this study was MACE, which comprised cardiac death, rehospitalization due to worsening HF, unplanned repeat revascularization, and non-fatal recurrent MI. Cardiac death was defined as death directly resulting from cardiac causes such as fatal arrhythmia, acute HF, or cardiogenic shock. Rehospitalization due to worsening HF was defined as an unplanned admission caused by exacerbation of typical HF symptoms (such as dyspnea, rapid weight gain, edema), with a diagnosis of HF confirmed by the treating physician. Non-fatal recurrent MI was defined according to the Fourth Universal Definition of Myocardial Infarction (2018), requiring both (1) a cardiac biomarker (such as cTnI or cTnT) level above the 99th percentile upper reference limit with a rising and/or falling pattern, and (2) clinical evidence of myocardial ischemia such as ischemic symptoms, new ST-segment changes or new left bundle branch block, pathological Q waves, imaging evidence of new loss of viable myocardium or regional wall motion abnormalities, or identification of a new coronary artery occlusion or thrombus by angiography (17). Unplanned repeat revascularization referred to non-scheduled PCI or coronary artery bypass grafting (CABG) procedures due to recurrent myocardial ischemia, confirmed by symptoms, ECG, or imaging findings. All MACE events were adjudicated based on data obtained through electronic medical records, discharge summaries, and post-discharge telephone follow-up. Each event was independently reviewed by two cardiologists, and discrepancies were resolved by a third senior cardiovascular expert.

### 2.4 Measurement of lipoprotein(a)

Lp(a) was measured via the immunoturbidimetric method via an AU5800 automatic biochemical analyser (Beckman Coulter, USA). Fasting venous blood samples were collected within 24 h of admission, typically in the early morning after an overnight fast. The Lp(a) assay was performed using an immunoturbidimetric method, which detects antigen–antibody complexes formed between Lp(a) and specific anti-Lp(a) antibodies. The reaction was monitored photometrically to quantify Lp(a) concentration. The normal reference range for Lp(a) is 0–300 mg/L. Patients were divided into three tertiles according to Lp(a) level: T1 (≤356 mg/L), T2 [356 mg/L < Lp(a) ≤ 487 mg/L], and T3 (>487 mg/L). Since Lp(a) levels in this study were not normally distributed, a Log_10_ transformation was applied to yield Log_10_Lp(a).

### 2.5 Statistical analysis

All the statistical analyses were performed using SPSS version 26.0 (SPSS Inc., Chicago, IL, USA). Categorical variables were expressed as frequencies and percentages. Continuous variables that followed a normal distribution were presented as the means ± standard deviations, whereas nonnormally distributed continuous variables were expressed as medians and interquartile ranges. Differences between groups were assessed using the chi-square test for categorical variables, the independent samples *t*-test for normally distributed continuous variables, and the Kruskal–Wallis test for nonnormally distributed continuous variables. Cox regression analyses were conducted to identify the association Lp(a) with MACE. To validate the use of Cox proportional hazards models, we tested the proportional hazards (PH) assumption for all included covariates. Time-dependent covariate analysis was performed by creating interaction terms between each predictor and the natural logarithm of survival time [log(time)]. Each interaction term was entered into the multivariate Cox model to assess whether the effect of each variable on the hazard ratio changed over time. None of the interaction terms reached statistical significance (all *P* > 0.05), indicating that the PH assumption was not violated. Subgroup analyses were performed to explore the potential effect modification of the association between Lp(a) and MACE. The ability of Lp(a) level to predict MACE was evaluated using receiver operating characteristic (ROC) curve analysis. Kaplan–Meier survival analysis was conducted to estimate the cumulative incidence of MACE across different Lp(a) tertile groups. Statistical significance was set at a two-sided *P*-value of <0.05.

## 3 Results

### 3.1 Baseline demographics

Table 1 presented the clinical characteristics of the population grouped based on MACE occurrence. Compared with the non-MACE group, the MACE group had a greater median age, and elevated levels of SBP, Lp(a), Log_10_Lp(a), D-dimer and BNP. Additionally, the MACE group had a higher prevalence of diabetes, AF, and CKD, and higher usage rates of hypoglycemic agents. These patients also exhibited a higher incidence of multivessel disease, as well as reduced eGFR (*P* < 0.05).

**Table 1:** Clinical characteristics according to MACE groups.

Variables	Total population	Non-MACE	MACE	*P*-value
Age, years	74.41 ± 11.16	71.82 ± 10.90	75.96 ± 11.04	<0.001
Male, *n* (%)	208 (52.1)	86 (57.3)	122 (49.0)	0.106
Smoking, *n* (%)	120 (30.1)	49 (32.7)	71 (28.5)	0.381
Drinking, *n* (%)	69 (17.3)	28 (18.7)	41 (16.5)	0.573
STEMI, *n* (%)	172 (43.1)	83 (55.3)	89 (35.7)	<0.001
Killip class, *n* (%)				
I	196 (49.1)	83 (55.3)	113 (45.4)	0.021
II	147 (36.8)	56 (37.3)	91 (36.5)	
III	32 (8.0)	7 (4.7)	25 (10.0)	
IV	24 (6.0)	4 (2.7)	20 (8.0)	
Family history of CHD, *n* (%)	61 (15.3)	23 (15.3)	38 (15.3)	0.984
Hypertension, *n* (%)	277 (69.4)	96 (64.0)	181 (72.7)	0.068
Diabetes, *n* (%)	253 (63.4)	85 (56.7)	168 (67.5)	0.030
Stroke, *n* (%)	53 (13.3)	18 (12.0)	35 (14.1)	0.558
Atrial fibrillation, *n* (%)	36 (9.0)	8 (5.3)	28 (11.2)	0.046
CKD, *n* (%)	164 (41.1)	50 (33.3)	114 (45.8)	0.014
Previous medicine, *n* (%)				
Antihypertensive drugs	277 (69.4)	96 (64.0)	181 (72.7)	0.068
Hypoglycemic agents	208 (52.1)	67 (44.7)	141 (56.6)	0.021
Lipid-lowering drugs	33 (8.3)	12 (8.0)	21 (8.4)	0.879
Body mass index, kg/m^2^	26.76 ± 3.96	26.91 ± 4.25	26.67 ± 3.78	0.563
SBP, mmHg	132.11 ± 29.63	128.07 ± 28.52	134.54 ± 30.07	0.034
DBP, mmHg	74.73 ± 15.32	74.55 ± 15.68	74.84 ± 15.14	0.855
Heart rate, bpm	80.97 ± 22.74	78.15 ± 20.61	82.68 ± 23.81	0.054
FPG, mmol/L	6.12 (5.17, 8.85)	6.15 (5.03, 8.14)	6.08 (5.17, 9.34)	0.147
HbA1c, %	7.20 (5.90, 8.80)	6.60 (5.80, 8.70)	7.50 (6.00, 8.70)	0.268
Triglycerides, mmol/L	1.28 (0.94, 1.84)	1.34 (0.98, 1.81)	1.21 (0.94, 1.84)	0.440
Total cholesterol, mmol/L	4.67 ± 1.29	4.72 ± 1.33	4.64 ± 1.26	0.571
LDL-C, mmol/L	2.92 ± 0.93	2.94 ± 0.89	2.91 ± 0.95	0.701
HDL-C, mmol/L	1.03 ± 0.29	1.03 ± 0.31	1.02 ± 0.28	0.676
Apolipoprotein A1, g/L	0.90 ± 0.21	0.89 ± 0.20	0.91 ± 0.21	0.593
Apolipoprotein B, g/L	1.05 ± 0.29	1.05 ± 0.29	1.06 ± 0.30	0.853
Lp(a), mg/L	393.00 (324.00, 539.00)	356.00 (280.00, 454.50)	427.00 (359.50, 556.50)	<0.001
Log_10_Lp(a)	2.58 ± 0.24	2.51 ± 0.25	2.62 ± 0.22	<0.001
Albumin, g/L	35.98 ± 3.98	36.19 ± 4.11	35.85 ± 3.91	0.413
Uric acid, umol/L	387.73 ± 127.97	393.71 ± 122.68	384.13 ± 131.17	0.470
eGFR, ml/min	68.00 (47.00, 85.00)	73.00 (54.00, 92.00)	67.00 (40.00, 85.00)	0.009
Hs-CRP, mg/L	44.70 (24.00, 88.80)	44.70 (23.55, 104.50)	48.20 (28.10, 97.00)	0.822
Fibrinogen, g/L	3.48 (2.72, 4.54)	3.27 (2.43, 4.33)	3.51 (2.79, 4.65)	0.065
D-dimer, mg/L	150.00 (0.71, 690.00)	120.00 (0.50, 420.00)	290.00 (0.76, 770.00)	0.012
Hs-cTnI, ng/ml	8.25 (1.50, 49.01)	21.96 (2.13, 117.08)	13.56 (2.12, 49.01)	0.135
BNP, pg/ml	695.55 (523.54, 1,085.32)	619.86 (509.56, 794.29)	691.53 (511.88, 1,057.98)	0.022
Echocardiography				
LVEF, %	54.72 ± 3.05	54.93 ± 3.01	54.60 ± 3.07	0.304
LAD, mm	39.43 ± 5.22	39.38 ± 5.59	39.46 ± 4.97	0.889
LVDD, mm	47.31 ± 4.74	47.73 ± 4.70	47.04 ± 4.77	0.204
IVSD, mm	10.94 ± 1.96	11.01 ± 1.95	10.89 ± 1.96	0.608
LVPW, mm	10.39 ± 1.28	10.37 ± 1.20	10.41 ± 1.33	0.811
Coronary angiography				
Multivessel disease, *n* (%)	189 (47.4)	59 (39.3)	130 (52.2)	0.013
Three-vessel disease, *n* (%)	61 (15.3)	19 (12.7)	42 (16.9)	0.259
Bracket length, mm	28.00 (21.00, 35.00)	25.00 (21.00, 33.00)	28.00 (21.00, 35.00)	0.163
Bracket diameter, mm	3.00 (2.50, 3.50)	3.00 (2.50, 3.50)	3.00 (2.50, 3.50)	0.996

MACE, major adverse cardiovascular events; STEMI, ST-elevation myocardial infarction; CHD, coronary heart disease; CKD, chronic kidney disease; SBP, systolic blood pressure; DBP, diastolic blood pressure; FPG, fasting plasma glucose; HbA1c, hemoglobin A1c; LDL-C, low-density lipoprotein cholesterol; HDL-C, high-density lipoprotein cholesterol; Lp(a), lipoprotein(a); eGFR, estimated glomerular filtration rate; Hs-CRP, high-sensitivity C-reactive protein; Hs-cTnI, high-sensitivity cardiac troponin I; BNP, B-type natriuretic peptide; LVEF, left ventricular ejection fraction; LAD, left atrial diameter; LVDD, left ventricular end-diastolic diameter; IVSD, interventricular septal diameter; LVPW, left ventricular posterior wall thickness.

Table 2 presented the clinical characteristics of the population grouped by Lp(a) tertiles. The results indicated statistically significant differences among the Lp(a) groups in terms of age, BMI, LDL-C, Hs-CRP, multivessel disease, and three-vessel disease. Moreover, across the Lp(a) tertile groups, the incidence of MACE, rehospitalization due to worsening HF, non-fatal recurrent MI, and unplanned repeat revascularization significantly increased with higher Lp(a) levels (all *P* < 0.05), whereas the difference in cardiac death was not statistically significant (*P* = 0.126).

**Table 2:** Clinical characteristics based on Lp(a) tertiles.

Variables	T1	T2	T3	*P*-value
Age, years	74.32 ± 11.83	76.22 ± 10.62	72.73 ± 10.78	0.039
Male, *n* (%)	64 (47.1)	72 (55.4)	72 (54.1)	0.338
Smoking, *n* (%)	37 (27.2)	39 (30.0)	44 (33.1)	0.567
Drinking, *n* (%)	25 (18.4)	22 (16.9)	22 (16.5)	0.915
STEMI, *n* (%)	60 (44.1)	55 (42.3)	57 (42.9)	0.954
Killip class, *n* (%)				
I	63 (46.3)	55 (42.3)	78 (58.6)	0.174
II	51 (37.5)	54 (41.5)	42 (31.6)	
III	13 (9.6)	13 (10.0)	6 (4.5)	
IV	9 (6.6)	8 (6.2)	7 (5.3)	
Family history of CHD, *n* (%)	25 (18.4)	18 (13.8)	18 (13.5)	0.465
Hypertension, *n* (%)	93 (68.4)	90 (69.2)	94 (70.7)	0.918
Diabetes, *n* (%)	91 (66.9)	81 (62.3)	81 (60.9)	0.563
Stroke, *n* (%)	16 (11.8)	17 (13.1)	20 (15.0)	0.729
Atrial fibrillation, *n* (%)	12 (8.8)	15 (11.5)	9 (6.8)	0.400
CKD, *n* (%)	49 (36.0)	58 (44.6)	57 (42.9)	0.320
Previous medicine, *n* (%)				
Antihypertensive drugs	93 (68.4)	90 (69.2)	94 (70.7)	0.918
Hypoglycemic agents	71 (52.2)	63 (48.5)	74 (55.6)	0.507
Lipid-lowering drugs	7 (5.1)	11 (8.5)	15 (11.3)	0.188
Body mass index, kg/m^2^	26.82 ± 4.12	26.05 ± 3.62	27.40 ± 4.02	0.021
SBP, mmHg	132.39 ± 30.30	130.37 ± 29.52	133.51 ± 29.17	0.685
DBP, mmHg	76.07 ± 14.82	72.28 ± 14.20	75.77 ± 16.67	0.083
Heart rate, bpm	80.14 ± 21.00	82.40 ± 24.96	80.44 ± 22.28	0.682
FPG, mmol/L	6.38 (4.95, 8.70)	5.89 (5.17, 9.09)	6.15 (5.15, 9.27)	0.915
HbA1c, %	7.40 (5.90, 8.90)	7.10 (5.90, 8.40)	6.90 (6.00, 9.03)	0.498
Triglycerides, mmol/L	1.34 (1.02, 1.86)	1.21 (0.91, 1.78)	1.30 (0.96, 1.86)	0.332
Total cholesterol, mmol/L	4.67 ± 1.33	4.58 ± 1.20	4.76 ± 1.32	0.496
LDL-C, mmol/L	3.09 ± 0.94	2.50 ± 0.77	3.15 ± 0.92	<0.001
HDL-C, mmol/L	1.01 ± 0.31	1.03 ± 0.27	1.03 ± 0.29	0.835
Apolipoprotein A1, g/L	0.88 ± 0.19	0.91 ± 0.21	0.91 ± 0.21	0.367
Apolipoprotein B, g/L	1.02 ± 0.28	1.09 ± 0.33	1.06 ± 0.26	0.124
Albumin, g/L	35.99 ± 4.25	36.11 ± 3.74	35.84 ± 3.95	0.860
Uric acid, umol/L	382.21 ± 131.28	394.56 ± 126.19	386.71 ± 126.94	0.730
eGFR, ml/min	68.00 (50.00, 86.00)	70.00 (49.50, 88.00)	69.50 (40.00, 87.75)	0.743
Hs-CRP, mg/L	44.70 (24.40, 99.00)	69.50 (44.40, 123.50)	35.75 (20.08, 66.05)	<0.001
Fibrinogen, g/L	3.32 (2.51, 4.56)	3.26 (2.55, 4.28)	3.63 (2.82, 4.72)	0.035
D dimer, mg/L	140.00 (0.53, 600.00)	310.00 (0.87, 840.00)	130.00 (0.63, 505.00)	0.406
Hs-cTnI, ng/ml	21.98 (1.91, 143.10)	14.28 (2.44, 52.72)	11.48 (2.05, 51.93)	0.723
BNP, pg/ml	645.32 (508.69, 844.08)	685.61 (527.92, 948.94)	654.15 (505.40, 1,016.48)	0.569
Echocardiography				
LVEF, %	54.76 ± 3.08	54.67 ± 2.92	54.74 ± 3.16	0.969
LAD, mm	39.79 ± 4.77	39.98 ± 6.33	38.53 ± 4.42	0.089
LVDD, mm	47.20 ± 4.56	47.77 ± 5.00	47.02 ± 4.71	0.505
IVSD, mm	11.13 ± 2.10	10.83 ± 2.08	10.83 ± 1.65	0.422
LVPW, mm	10.39 ± 1.22	10.32 ± 1.34	10.47 ± 1.30	0.702
Coronary angiography				
Multivessel disease, *n* (%)	36 (26.5)	52 (40.0)	101 (75.9)	<0.001
Three-vessel disease, *n* (%)	13 (9.6)	12 (9.2)	36 (27.1)	<0.001
Bracket length, mm	25.00 (23.00, 35.00)	25.00 (21.00, 35.00)	29.00 (19.00, 35.00)	0.996
Bracket diameter, mm	3.00 (2.50, 3.50)	3.00 (2.50, 3.50)	3.00 (2.75, 3.50)	0.327
MACE, *n* (%)	60 (44.1)	89 (68.5)	100 (75.2)	<0.001
Cardiac death	25 (18.4)	34 (26.2)	38 (28.6)	0.126
Rehospitalization due to worsening HF	35 (25.7)	45 (34.6)	63 (47.4)	0.001
Unplanned repeat revascularization	14 (10.3)	22 (16.9)	34 (25.6)	0.004
Non-fatal recurrent MI	11 (8.1)	16 (12.3)	33 (24.8)	<0.001

Lp(a), lipoprotein(a); STEMI, ST-elevation myocardial infarction; CHD, coronary heart disease; CKD, chronic kidney disease; SBP, systolic blood pressure; DBP, diastolic blood pressure; FPG, fasting plasma glucose; HbA1c, hemoglobin A1c; LDL-C, low-density lipoprotein cholesterol; HDL-C, high-density lipoprotein cholesterol; eGFR, estimated glomerular filtration rate; Hs-CRP, high-sensitivity C-reactive protein; Hs-cTnI, high-sensitivity cardiac troponin I; BNP, B-type natriuretic peptide; LVEF, left ventricular ejection fraction; LAD, left atrial diameter; LVDD, left ventricular end-diastolic diameter; IVSD, interventricular septal diameter; LVPW, left ventricular posterior wall thickness; MACE, major adverse cardiovascular events; HF, heart failure; MI, myocardial infarction.

### 3.2 Correlation between Lp(a) and MACE

Supplementary Table S1 showed the univariate Cox regression analysis for MACE, indicating that age, hypertension, diabetes, AF, CKD, antihypertensive drugs, hypoglycemic agents, SBP, Lp(a), Log_10_Lp(a), eGFR, BNP, and multivessel disease were all significantly associated with MACE occurrence (*P* < 0.05).

Table 3 presented the multivariate Cox regression analysis for Lp(a) and MACE. The results showed that in the unadjusted Model 1 and the partially adjusted Model 2 (which accounted for age, hypertension, diabetes, AF, CKD, antihypertensive, and hypoglycemic agents), Lp(a) was significantly associated with MACE, whether treated as a categorical or continuous variable (*P* < 0.05). In the fully adjusted Model 3 (which was further adjusted for age, hypertension, diabetes, AF, CKD, antihypertensives, hypoglycemic agents, SBP, FPG, and multivessel disease), Lp(a) remained significantly associated with MACE (*P* < 0.05). When treated as a categorical variable, the risk of MACE in the T2 and T3 groups was 1.642 and 2.195 times higher, respectively, than that in the T1 group (*P* < 0.05). When Lp(a) levels were ≥300 mg/L, the risk of MACE was 2.088 times greater than that when Lp(a) levels were <300 mg/L, and when Lp(a) levels were ≥500 mg/L, the risk was 1.611 times greater than that when Lp(a) levels were <500 mg/L (*P* < 0.05). When treated as a continuous variable, for every unit increase in Lp(a) level, the risk of MACE increased by 0.1%, and for every unit increase in Log_10_Lp(a), the risk of MACE increased by 270.3% (*P* < 0.05). In Additional Model 4, after adjusting for age, STEMI, Killip class, hypertension, diabetes, AF, antihypertensive drugs, antidiabetic drugs, SBP, FPG, eGFR, BNP, and multivessel disease, Lp(a) remained an independent predictor of MACE, with significantly elevated HRs across both categorical and continuous variable analyses (all *P* < 0.05).

**Table 3:** The multivariate Cox regression analysis of Lp(a) and MACE.

Variables	Model 1			Model 2			Model 3			Additional Model 4		
	HR	95% CI	*P*-value	HR	95% CI	*P*-value	HR	95% CI	*P*-value	HR	95% CI	*P*-value
Categorical variable												
T1	Ref			Ref			Ref			Ref		
T2	1.778	1.281–2.468	0.001	1.642	1.180–2.285	0.003	1.642	1.180–2.285	0.003	1.665	1.197–2.317	0.002
T3	2.151	1.559–2.967	<0.001	2.195	1.590–3.029	<0.001	2.195	1.590–3.029	<0.001	2.359	1.704–3.266	<0.001
Lp(a) <300	Ref			Ref			Ref			Ref		
Lp(a) ≥300	2.148	1.430–3.226	<0.001	2.026	1.348–3.046	0.001	2.088	1.388–3.142	<0.001	1.883	1.234–2.872	0.003
Lp(a) <500	Ref			Ref			Ref			Ref		
Lp(a) ≥500	1.497	1.158–1.936	0.002	1.611	1.243–2.089	<0.001	1.611	1.243–2.089	<0.001	1.701	1.307–2.215	<0.001
Continuous variable												
Lp(a)	1.001	1.001–1.002	<0.001	1.001	1.001–1.002	<0.001	1.001	1.001–1.002	<0.001	1.002	1.001–1.002	<0.001
Log_10_Lp(a)	3.678	1.933–6.997	<0.001	3.703	1.901–7.214	<0.001	3.703	1.901–7.214	<0.001	4.250	2.157–8.374	<0.001

Model 1: Unadjusted; Model 2: Adjusted for age, hypertension, diabetes, AF, CKD, antihypertensive drugs, and antidiabetic drugs; Model 3: Adjusted for age, hypertension, diabetes, AF, CKD, antihypertensive drugs, hypoglycemic agents, SBP, fasting plasma glucose, multivessel disease; Additional Model 4: Adjusted for age, STEMI, Killip class, hypertension, diabetes, AF, CKD, antihypertensive drugs, hypoglycemic agents, SBP, fasting plasma glucose, eGFR, BNP, and multivessel disease. Lp(a), lipoprotein(a); MACE, major adverse cardiovascular events; AF, atrial fibrillation; CKD, chronic kidney disease; STEMI, ST-segment elevation myocardial infarction; SBP, systolic blood pressure; eGFR, estimated glomerular filtration rate; BNP, B-type natriuretic peptide; HR, hazard ratio; CI, confidence interval.

Supplementary Table S2 showed that elevated levels of Lp(a) were significantly associated with several components of MACE. Specifically, patients in the highest Lp(a) tertile (T3) had a significantly increased risk of cardiac death (HR = 2.106, *P* = 0.004), and Log_10_Lp(a) was also significantly associated (HR = 3.266, *P* = 0.025). For rehospitalization due to worsening HF, the risk was significantly higher in the T3 group (HR = 2.559, *P* < 0.001), and both Lp(a) as a continuous variable (HR = 1.002, *P* < 0.001) and Log_10_Lp(a) (HR = 3.347, *P* = 0.015) demonstrated independent predictive value. Additionally, Lp(a) was significantly associated with non-fatal recurrent MI, with consistent results observed for both continuous Lp(a) (HR = 1.002, *P* = 0.022) and Log_10_Lp(a) (HR = 6.609, *P* = 0.033). In contrast, although Lp(a) showed an upward trend in relation to unplanned repeat revascularization, the association did not reach statistical significance.

### 3.3 Subgroup analysis

In the subgroup analysis presented in Table 4, elevated Lp(a) levels were significantly associated with an increased risk of MACE across a wide range of subgroups. This association was observed in patients aged <75 years (T2 vs. T1: HR = 2.082, *P* = 0.007; T3 vs. T1: HR = 2.726, *P* < 0.001) and ≥75 years (T3 vs. T1: HR = 1.921, *P* = 0.004); in males (T2 vs. T1: HR = 2.577, *P* < 0.001; T3 vs. T1: HR = 3.119, *P* < 0.001) and females (T3 vs. T1: HR = 1.495, *P* = 0.010); in patients with STEMI (T2 vs. T1: HR = 2.185, *P* < 0.001; T3 vs. T1: HR = 3.005, *P* < 0.001) and without STEMI (T3 vs. T1: HR = 1.916, *P* < 0.001). Similar significant associations were found in those with hypertension (T2 vs. T1: HR = 1.692, *P* = 0.007; T3 vs. T1: HR = 2.200, *P* < 0.001) and without hypertension (T3 vs. T1: HR = 2.451, *P* = 0.006), with diabetes (T2 vs. T1: HR = 1.720, *P* = 0.008; T3 vs. T1: HR = 2.414, *P* < 0.001) and without diabetes (T3 vs. T1: HR = 2.238, *P* = 0.005), as well as in patients with LDL-C ≥4.1 mmol/L (T3 vs. T1: HR = 4.059, *P* = 0.037) and <4.1 mmol/L (T2 vs. T1: HR = 1.665, *P* = 0.004; T3 vs. T1: HR = 2.383, *P* < 0.001). Furthermore, Lp(a) remained a strong predictor among patients with CKD (T2 vs. T1: HR = 2.430, *P* = 0.001; T3 vs. T1: HR = 2.731, *P* < 0.001) and without CKD (T3 vs. T1: HR = 1.779, *P* = 0.007), as well as among those with BMI <26.77 kg/m^2^ (T3 vs. T1: HR = 2.164, *P* = 0.001) and ≥26.77 kg/m^2^ (T2 vs. T1: HR = 1.894, *P* = 0.011; T3 vs. T1: HR = 2.245, *P* = 0.001).

**Table 4:** The subgroup association between Lp(a) and MACE.

Subgroups	T1		T2		T3		*P* for trend
	HR (95% CI)	*P*	HR (95% CI)	*P*	HR (95% CI)	*P*	
Age							
<75 years (*n* = 196)	Ref		2.082 (1.223–3.545)	0.007	2.726 (1.665–4.464)	<0.001	<0.001
≥75 years (*n* = 203)	Ref		1.209 (0.788–1.853)	0.385	1.921 (1.235–2.986)	0.004	0.012
Sex							
Male (*n* = 208)	Ref		2.577 (1.536–4.322)	<0.001	3.119 (1.889–5.149)	<0.001	<0.001
Female (*n* = 191)	Ref		1.026 (0.634–1.659)	0.917	1.495 (0.913–2.448)	0.110	0.170
STEMI							
Yes (*n* = 172)	Ref		2.185 (1.222–3.907)	0.008	3.005 (1.695–5.329)	<0.001	<0.001
No (*n* = 227)	Ref		1.449 (0.967–2.172)	0.072	1.916 (1.291–2.844)	0.001	0.005
Hypertension							
Yes (*n* = 277)	Ref		1.692 (1.153–2.484)	0.007	2.200 (1.503–3.221)	<0.001	<0.001
No (*n* = 122)	Ref		1.784 (0.920–3.460)	0.087	2.451 (1.290–4.655)	0.006	0.018
Diabetes							
Yes (*n* = 253)	Ref		1.720 (1.152–2.569)	0.008	2.414 (1.613–3.614)	<0.001	<0.001
No (*n* = 146)	Ref		1.326 (0.716–2.455)	0.369	2.238 (1.272–3.936)	0.005	0.011
Dyslipidemia							
LDL-C≥ 4.1 mmol/L (*n* = 37)	Ref		0.745 (0.056–9.893)	0.823	4.059 (1.086–15.169)	0.037	0.064
LDL-C <4.1 mmol/L (*n* = 362)	Ref		1.665 (1.180–2.349)	0.004	2.383 (1.679–3.381)	<0.001	<0.001
CKD							
Yes (*n* = 164)	Ref		2.430 (1.446–4.083)	0.001	2.731 (1.627–4.584)	<0.001	<0.001
No (*n* = 235)	Ref		1.218 (0.786–1.886)	0.377	1.779 (1.172–2.701)	0.007	0.021
BMI							
<26.77 kg/m^2^ (*n* = 200)	Ref		1.420 (0.908–2.219)	0.124	2.164 (1.380–3.395)	0.001	0.003
≥26.77 kg/m^2^ (*n* = 199)	Ref		1.894 (1.155–3.106)	0.011	2.245 (1.401–3.597)	0.001	0.002

The subgroup analysis was adjusted for age, hypertension, diabetes, AF, CKD, antihypertensive drugs, hypoglycemic agents, SBP, fasting plasma glucose, multivessel disease. Lp(a), lipoprotein(a); MACE, major adverse cardiovascular events; AF, atrial fibrillation; LDL-C, low-density lipoprotein cholesterol; CKD, chronic kidney disease; STEMI, ST-segment elevation myocardial infarction; BMI, body mass index; SBP, systolic blood pressure; HR, hazard ratio; CI, confidence interval.

### 3.4 ROC curve and Kaplan–Meier survival analysis

**Figure 2:**
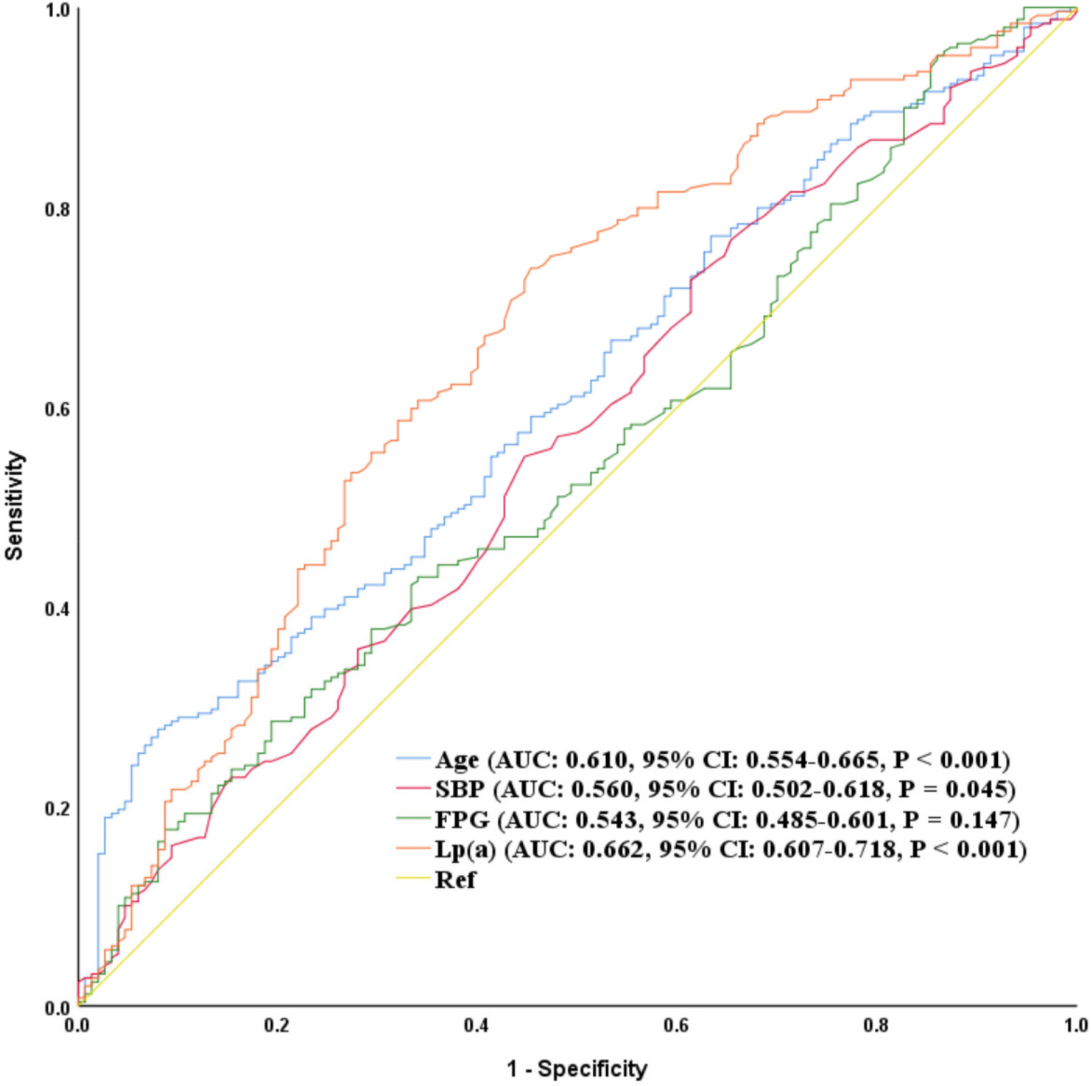
Receiver operating characteristic curve analysis of Lp(a) for predicting MACE. Lp(a), lipoprotein(a); SBP, systolic blood pressure; FPG, fasting plasma glucose; AUC, area under the curve; CI, confidence interval; MACE, major adverse cardiovascular events.

**Figure 3:**
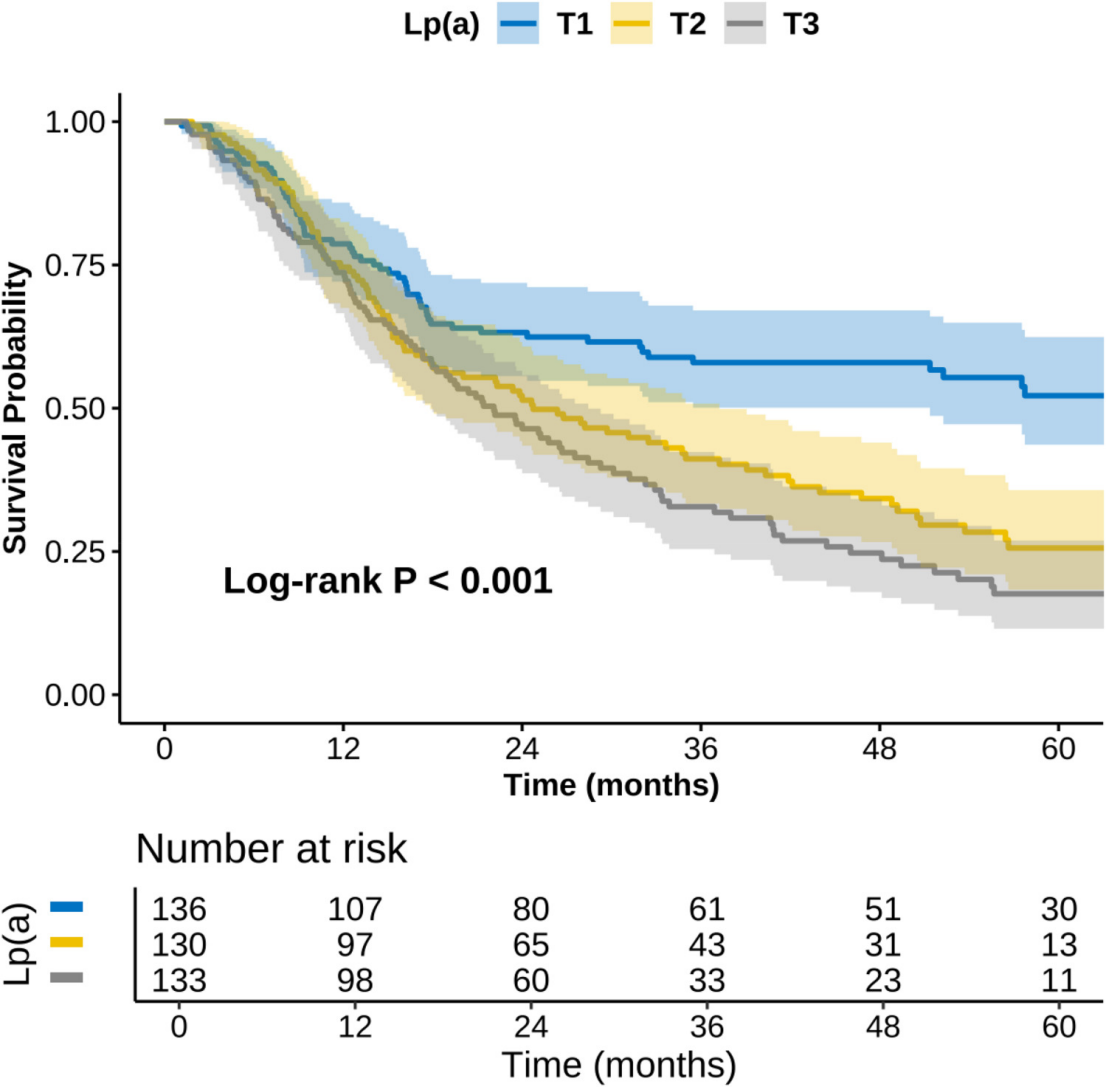
Kaplan–Meier survival curve of MACE stratified by Lp(a) tertiles. Lp(a), lipoprotein(a); MACE, major adverse cardiovascular events.

Figure 2 and Supplementary Table S3 showed that the area under the receiver operating characteristic curve (AUC) for Lp(a) in predicting MACE was 0.662 (95% CI: 0.607–0.718, *P* < 0.001), with a sensitivity of 73.9% and a specificity of 54.7%, which was significantly higher than that of SBP (AUC = 0.560, *P* = 0.045) and FPG (AUC = 0.543, *P* = 0.147). However, the difference compared to age (AUC = 0.610, *P* < 0.001) was not statistically significant (*P* for comparison = 0.211). These results suggest that Lp(a) has good discriminatory ability in predicting MACE and outperforms certain traditional cardiovascular risk factors. As illustrated in Figure 3, the Kaplan–Meier survival curve indicated that the survival probability without MACE significantly differed across the three Lp(a) groups over the follow-up period (*P* < 0.001), with the higher Lp(a) groups showing the fastest decline in survival probability and the poorest prognosis.

## 4 Discussion

This study systematically investigated the association between Lp(a) and MACE in patients with AMI complicated by HFpEF who underwent PCI. The results showed that the incidence of MACE significantly increased with rising Lp(a) levels, and multivariate Cox regression analysis confirmed that Lp(a) remained an independent predictor of MACE across multiple adjusted models. This trend was consistently observed across various clinical subgroups, including age (<75 or ≥75 years), male, STEMI status (yes or no), hypertension (yes or no), diabetes (yes or no), CKD (yes or no), LDL-C levels (≥4.1 mmol/L or <4.1 mmol/L), and BMI (<26.77 kg/m^2^ or ≥26.77 kg/m^2^), indicating the robustness of the association in different patient populations. No statistically significant interactions were found, suggesting that the predictive value of Lp(a) was not substantially modified by these stratifying factors. Further analysis revealed that elevated Lp(a) levels were significantly associated with several components of MACE, including rehospitalization due to worsening HF, non-fatal recurrent MI, and cardiac death, with the highest risk observed in the T3 group. Importantly, we found that Lp(a) ≥300 mg/L was associated with a markedly higher risk of MACE, and 82.5% of patients in our cohort exceeded this threshold, suggesting that 300 mg/L may serve as a more clinically representative and practical cutoff for identifying high-risk individuals. These findings suggest a warning role of Lp(a) in different adverse events. Moreover, Kaplan–Meier survival curves and ROC analysis further supported the predictive value of Lp(a) for MACE, demonstrating better discriminatory ability than some traditional cardiovascular risk factors, such as SBP and FPG. Collectively, these findings highlight the potential of Lp(a) as a promising biomarker for early risk stratification and precision intervention in high-risk AMI patients with HFpEF.

Left ventricular (LV) dilation and dysfunction caused by ischemic heart disease, which refers to structural and functional remodelling of the LV, can lead to a decreased LVEF or hemodynamic abnormalities. However, the widespread use of PCI has led to patients experiencing mitigation and even reversal of this type of heart dysfunction caused by CHD and microvascular dysfunction. These patients are classified as having HFpEF caused by either large vessel obstruction or microvascular dysfunction (20–22). Recently, an increasing number of scholars have focused on the relationship between metabolic disorders and HFpEF, particularly in the area of lipid metabolism, where research is currently limited (23). As mentioned earlier, Lp(a) is an independent risk factor for CVD, with concentrations exceeding 30 mg/dl accelerating atherosclerosis. Current research on Lp(a) management focuses primarily on lipid metabolism, CHD, and the efficacy of related lipid-lowering drugs. While therapies such as niacin or PCSK9 inhibitors can lower Lp(a) levels, they have not been shown to significantly reduce the cardiovascular event risk associated with Lp(a). There is currently limited research and attention on ischemic heart disease patients with HF after MI, particularly regarding the HFpEF type. In our study, after adjusting for relevant variables, the results indicated that Lp(a) remained an independent predictor of MACE in the AMI population with HFpEF. Although previous studies have not extensively explored the correlation between Lp(a) and HFpEF induced by ischemia, some research has indicated that Lp(a) is related to the risk of CHD or AMI (24). Al Hageh et al. identified that Lp(a) level could serve as a primary marker for severe and multiple stenosis lesions, with a positive correlation between high Lp(a) levels and stenosis in the RCA and LCX (25). Notably, even when considering Lp(a) levels ≥30 mg/dl, the risk of stenosis significantly increased with increasing Lp(a) levels. This correlation is consistent with some of our findings, supporting the notion that higher Lp(a) levels are correlated with a higher incidence of multivessel disease, three-vessel disease, LM, and LCX lesions, thus increasing the risk of HF and MACE. Bittner et al. reported that baseline levels of Lp(a) and LDL-C, along with the effects of PCSK9 inhibitors on their reduction, could predict the risk of MACE shortly after acute coronary syndrome (ACS). These findings suggest that Lp(a) may be an important independent therapeutic target in ACS to reduce the likelihood of MACE and improve long-term survival (26).

Previous studies have also addressed the relationship between lipid metabolism and HF following MI. Da Dalt et al. found that the development of HF was linked to cardiac lipid metabolism and mitochondrial dysfunction, with changes in primary energy substrates leading to an imbalance between fatty acid uptake and oxidation and resulting in lipid accumulation and mitochondrial dysfunction, ultimately causing HF (27). And Hahn et al. discovered a close relationship between myocardial metabolomics and HFpEF, indicating that, despite the presence of significant obesity and diabetes, the HFpEF myocardium presented lower fatty acid metabolites than did the HFrEF myocardium. This suggests insufficient utilization of alternative fuels, challenging traditional views on myocardial fuel utilization in HF patients with significant diabetes and obesity (28). Besides, Tsuda et al. found that a diminished response to statin therapy could predict the onset of HF after AMI, with a low response to statins being associated with an increased likelihood of AF and HF (29). Overall, while Lp(a) level can predict the incidence and mortality of various diseases, including HF, its correlation with ischemia-induced HFpEF remains unknown. Our study not only confirmed the association between Lp(a) and MACE occurrence in patients with AMI and HFpEF but also demonstrated a stratified association across multiple subgroups, showing a stable correlation among various sensitive populations. However, the relatively small sample sizes in certain subgroups may have limited the statistical power of the interaction tests. Therefore, the interpretation of subgroup interactions should be made with caution to avoid overestimation of subgroup-specific effects.

Importantly, Lp(a) is a significant component of lipid metabolism research, with elevated levels associated with increased CVD risk, and it also has prognostic value for secondary prevention of ACS in patients undergoing PCI. Although further prospective studies are needed to validate these findings, Lp(a) appears to be an independent predictor of left ventricular hypertrophy, left ventricular systolic dysfunction and chamber dilation-related HF events (30). The mechanisms by which Lp(a) leads to MACE in patients with AMI and HFpEF, as summarised in the relevant literature, include the following: (1) promoting oxidative stress, thereby accelerating atherosclerosis, leading to ventricular remodelling and HF, and consequently resulting in MACE (31); (2) being closely related to left ventricular hypertrophy and the degree of coronary artery stenosis, thus increasing the risk of rehospitalization or recurrent MI due to HFpEF symptoms after PCI (8, 30); and (3) inducing inflammation; the apolipoprotein(a) contained in Lp(a) resembles the structure of plasminogen and competitively binds to plasminogen receptors, obstructing its conversion to plasmin, which leads to thrombus formation and occlusion of large coronary vessels and microcirculation. In the context of HFpEF, Lp(a) may also contribute to adverse events through additional biological mechanisms. First, its pro-atherogenic and pro-inflammatory properties may exacerbate vascular calcification and coronary plaque instability, triggering recurrent MI (5, 32–35). Second, Lp(a)'s antifibrinolytic activity due to its structural similarity to plasminogen increases the likelihood of thrombotic events, including coronary re-occlusion or restenosis after PCI (5, 36). Third, in the pro-inflammatory and oxidative stress environment of HFpEF, Lp(a) may accelerate myocardial interstitial fibrosis and impair ventricular compliance, thereby increasing the risk of symptom recurrence and HF-related rehospitalization (37, 38). Therefore, Lp(a) can promote adverse cardiovascular events either independently or in conjunction with inflammation (39–41). In conclusion, although high-quality large-scale multicenter trials are needed to confirm these findings, the observation that patients with lower Lp(a) levels have a lower risk of MACE in HFpEF is consistent with our study results.

## 5 Study limitations

This study had several limitations. First, owing to its retrospective design, both selection bias and information bias were likely unavoidable. Although we adjusted for multiple covariates in the multivariate Cox regression models, residual confounding may still exist due to unmeasured variables such as medication adherence, lifestyle factors, or socioeconomic status. Second, HFpEF was primarily diagnosed using transthoracic echocardiography, which does not include parameters such as global longitudinal strain. Compared with exercise stress echocardiography, transthoracic echocardiography has lower sensitivity, potentially leading to missed diagnoses. Third, some survival analysis indicators in both patient groups lacked statistical significance, possibly due to the small sample size and the single-center design of the study. Fourth, the unit of measurement for Lp(a) level in this study was mg/L, which differs from the more commonly used mg/dl in international standards, highlighting the need for future improvements. Fifth, we did not specifically differentiate between the types of hypoglycemic agents and antihypertensive drugs used in the study, which may have affected the outcomes. Additionally, some diabetic patients may have been treated with sodium-glucose cotransporter-2 inhibitors and gastric inhibitory polypeptide/glucagon-like peptide-1 receptor agonists, which have demonstrated cardiovascular protective effects, but their specific effects were not considered in this analysis. Future research should focus on the impact of these drugs on MACE in patients with HFpEF. Sixth, although Lp(a) was significantly associated with MACE, the AUC was only 0.662, indicating a modest discriminative ability. This suggests that while Lp(a) may serve as a useful biomarker for risk stratification, its predictive power alone is limited. Future studies may consider combining Lp(a) with other clinical indicators or biomarkers to improve predictive accuracy. Seventh, stroke was not included as a study endpoint. Although stroke is a relevant cardiovascular event, our study focused on atherosclerotic outcomes commonly included in MACE definitions, namely, cardiac death, HF rehospitalization, non-fatal recurrent MI, and unplanned revascularization. The exclusion of stroke may limit the comprehensiveness of event assessment, and future research should consider its inclusion to provide a more holistic understanding of Lp(a)'s impact. Finally, the study population was drawn from a single tertiary care center, and only patients who underwent PCI were included. This may introduce regional and treatment-related selection bias and limit the generalizability of the findings to broader populations, especially those receiving conservative or alternative treatment strategies. Future prospective, multicenter studies are needed to validate and extend these findings in more diverse clinical settings.

## 6 Conclusions

In conclusion, this study confirms that elevated levels of Lp(a) significantly increase the risk of MACE in patients with AMI complicated by HFpEF, and this association remains consistent across a variety of clinical subgroups. Notably, Lp(a) demonstrated particularly strong predictive value for non-fatal recurrent MI and rehospitalization due to worsening HF among the MACE components. Given the consistent association observed in this study, routine Lp(a) testing may have clinical implications in patients with AMI and HFpEF. Early measurement of Lp(a) upon admission could help identify individuals at higher risk for adverse outcomes, thereby supporting more targeted surveillance and personalized management. This is particularly relevant for HFpEF populations, where effective risk stratification tools remain limited. Future prospective studies with larger sample sizes are warranted to validate the robustness and generalizability of these findings, especially those conducted across multiple centers and diverse populations to enhance external validity and causal inference. In addition, randomized clinical trials targeting Lp(a), such as using PCSK9 inhibitors or RNA interference based therapies, are needed to determine whether lowering Lp(a) levels can improve long-term outcomes in patients with AMI and HFpEF.

## Data Availability

The original contributions presented in the study are included in the article/Supplementary Material, further inquiries can be directed to the corresponding authors.
