# Supplementary material for: The correlation between lipoprotein(a) and major adverse cardiovascular events in patients with acute myocardial infarction combined with heart failure with preserved ejection fraction
Source: Front Cardiovasc Med. 2025 Jun 9;12:1515916. doi: 10.3389/fcvm.2025.1515916 (PMC12183255; doi:10.3389/fcvm.2025.1515916)
Supplement: Supplementary file 1 [file Datasheet1.docx]

| **Table S1.** The univariate Cox regression analysis of MACE. | | | |
| --- | --- | --- | --- |
| Variables | HR | 95% CI | P value |
| Age | 1.027 | 1.014-1.040 | < 0.001 |
| Male | 0.814 | 0.635-1.045 | 0.106 |
| Smoking | 1.168 | 0.887-1.538 | 0.269 |
| Drinking | 1.175 | 0.841-1.643 | 0.345 |
| STEMI | 1.666 | 1.284-2.160 | < 0.001 |
| Killip class |  |  |  |
| I | Ref |  |  |
| II | 1.199 | 0.910-1.581 | 0.197 |
| III | 1.641 | 1.063-2.533 | 0.025 |
| IV | 2.499 | 1.550-4.027 | < 0.001 |
| Family history of coronary heart disease |  |  |  |
| Hypertension | 1.382 | 1.046-1.827 | 0.023 |
| Diabetes | 1.353 | 1.038-1.764 | 0.026 |
| Stroke | 1.228 | 0.859-1.756 | 0.260 |
| Atrial fibrillation | 1.490 | 1.005-2.209 | 0.047 |
| Chronic kidney disease | 1.491 | 1.161-1.913 | 0.002 |
| Previous medicine |  |  |  |
| Antihypertensive drugs | 1.382 | 1.046-1.827 | 0.023 |
| Hypoglycemic agents | 1.423 | 1.107-1.829 | 0.006 |
| Lipid-lowering drugs | 1.113 | 0.712-1.741 | 0.638 |
| Body mass index | 0.996 | 0.966-1.026 | 0.785 |
| Systolic blood pressure | 1.006 | 1.002-1.010 | 0.003 |
| Diastolic blood pressure | 1.001 | 0.993-1.009 | 0.810 |
| Heart rate | 1.005 | 0.999-1.010 | 0.091 |
| Fasting plasma glucose | 1.024 | 1.000-1.048 | 0.048 |
| Hemoglobin A1c | 1.023 | 0.962-1.088 | 0.475 |
| Triglycerides | 1.026 | 0.903-1.166 | 0.690 |
| Total cholesterol | 0.960 | 0.868-1.061 | 0.425 |
| LDL-C | 0.982 | 0.855-1.127 | 0.792 |
| HDL-C | 0.915 | 0.599-1.398 | 0.682 |
| Apolipoprotein A1 | 1.089 | 0.607-1.953 | 0.775 |
| Apolipoprotein B | 0.923 | 0.598-1.425 | 0.719 |
| Lp(a) | 1.001 | 1.001-1.002 | < 0.001 |
| Log_10_Lp(a) | 3.678 | 1.933-6.997 | < 0.001 |
| Albumin | 0.987 | 0.957-1.019 | 0.427 |
| Uric acid | 1.000 | 0.999-1.001 | 0.532 |
| eGFR | 0.993 | 0.989-0.998 | 0.002 |
| Hs-CRP | 1.000 | 0.998-1.003 | 0.806 |
| Fibrinogen | 1.045 | 0.958-1.140 | 0.325 |
| D-dimer | 1.000 | 1.000-1.000 | 0.300 |
| Hs-cTnI | 0.999 | 0.998-1.000 | 0.057 |
| BNP | 1.000 | 1.000-1.000 | 0.001 |
| Echocardiography |  |  |  |
| LVEF | 0.982 | 0.944-1.022 | 0.377 |
| LAD, mm | 1.003 | 0.977-1.030 | 0.797 |
| LVDD, mm | 0.981 | 0.953-1.011 | 0.210 |
| IVSD, mm | 0.974 | 0.905-1.050 | 0.495 |
| LVPW, mm | 1.004 | 0.898-1.122 | 0.949 |
| Coronary angiography |  |  |  |
| Multivessel disease | 1.342 | 1.046-1.721 | 0.021 |
| Three-vessel disease | 1.204 | 0.864-1.678 | 0.274 |
| Bracket length | 1.006 | 0.986-1.027 | 0.533 |
| Bracket diameter | 1.010 | 0.759-1.343 | 0.948 |

MACE, major adverse cardiovascular events; STEMI, ST-segment elevation myocardial infarction; LDL-C, low-density lipoprotein cholesterol; HDL-C, high-density lipoprotein cholesterol; Lp(a), lipoprotein(a); eGFR, estimated glomerular filtration rate; Hs-CRP, high-sensitivity C-reactive protein; Hs-cTnI, high-sensitivity cardiac troponin I; BNP, B-type natriuretic peptide; LVEF, left ventricular ejection fraction; LAD, left atrial diameter; LVDD, left ventricular diastolic diameter; IVSD, interventricular septal diameter; LVPW, left ventricular posterior wall.

| **Table S2.** The multivariate Cox regression analysis of Lp(a) and cardiac death, rehospitalization due to worsening HF, unplanned repeat revascularization, and non-fatal recurrent MI. | | | | | | | | | | | | |
| --- | --- | --- | --- | --- | --- | --- | --- | --- | --- | --- | --- | --- |
|  | Cardiac death | | | Rehospitalization due to worsening HF | | | Unplanned repeat revascularization | | | Non-fatal recurrent MI | | |
| Variables | HR | 95% CI | P value | HR | 95% CI | P value | HR | 95% CI | P value | HR | 95% CI | P value |
| Categorical variable |  |  |  |  |  |  |  |  |  |  |  |  |
| T1 | Ref |  |  | Ref |  |  | Ref |  |  | Ref |  |  |
| T2 | 1.257 | 0.742-2.128 | 0.395 | 1.301 | 0.832-2.034 | 0.248 | 1.369 | 0.678-2.763 | 0.381 | 1.188 | 0.535-2.636 | 0.672 |
| T3 | 2.106 | 1.262-3.515 | 0.004 | 2.559 | 1.682-3.895 | < 0.001 | 1.701 | 0.847-3.418 | 0.136 | 1.822 | 0.856-3.878 | 0.119 |
| Lp(a) < 300 | Ref |  |  | Ref |  |  | Ref |  |  | Ref |  |  |
| Lp(a) ≥ 300 | 1.472 | 0.765-2.833 | 0.247 | 1.221 | 0.715-2.085 | 0.465 | 0.774 | 0.373-1.606 | 0.492 | 1.314 | 0.506-3.414 | 0.574 |
| Lp(a) < 500 | Ref |  |  | Ref |  |  | Ref |  |  | Ref |  |  |
| Lp(a) ≥ 500 | 1.639 | 1.064-2.525 | 0.025 | 1.796 | 1.239-2.601 | 0.002 | 1.301 | 0.766-2.211 | 0.330 | 1.539 | 0.871-2.718 | 0.137 |
| Continuous variable |  |  |  |  |  |  |  |  |  |  |  |  |
| Lp(a) | 1.002 | 1.001-1.003 | 0.002 | 1.002 | 1.001-1.003 | < 0.001 | 1.001 | 1.000-1.003 | 0.136 | 1.002 | 1.000-1.003 | 0.022 |
| Log_10_Lp(a) | 3.266 | 1.162-9.179 | 0.025 | 3.347 | 1.270-8.822 | 0.015 | 2.125 | 0.524-8.617 | 0.291 | 6.609 | 1.161-37.603 | 0.033 |

The multivariate Cox regression analysis adjusted for age, hypertension, diabetes, AF, CKD, antihypertensive drugs, hypoglycemic agents, SBP, fasting plasma glucose, multivessel disease. Lp(a), lipoprotein(a); MACE, major adverse cardiovascular events; AF, atrial fibrillation; CKD, chronic kidney disease; STEMI, ST-segment elevation myocardial infarction; SBP, systolic blood pressure; eGFR, estimated glomerular filtration rate; HF, heart failure; MI, myocardial infarction; HR, hazard ratio; CI, confidence interval.

| **Table S3.** Comparison of the predictive performance of Lp(a) and other cardiovascular risk factors for MACE. | | | | | | |
| --- | --- | --- | --- | --- | --- | --- |
|  | AUC | 95% CI | P value | Sensitivity | Specitivity | P for comparison |
| Lp(a) | 0.662 | 0.607-0.718 | < 0.001 | 73.9% | 54.7% | Ref |
| Age | 0.610 | 0.554-0.665 | < 0.001 | 27.7% | 92.0% | 0.211 |
| SBP | 0.560 | 0.502-0.618 | 0.045 | 76.7% | 34.7% | 0.012 |
| FPG | 0.543 | 0.485-0.601 | 0.147 | 28.5% | 80.7% | 0.004 |

Lp(a), lipoprotein(a); SBP, systolic blood pressure; FPG, fasting plasma glucose; AUC, area under the curve; CI, confidence interval; MACE, major adverse cardiovascular events.
